# Supplementary material for: Are carbon emissions trading and green financial instruments synergistic? -Comprehensive quantitative research based on content analysis
Source: PLoS One. 2024 Mar 7;19(3):e0298601. doi: 10.1371/journal.pone.0298601 (PMC10919601; doi:10.1371/journal.pone.0298601)
Supplement: S1 Data — (ZIP) [file pone.0298601.s001.zip › Data/地级市Z_F工作报告.zip!/╜¡╬≈/╔╧╚─╩╨/└┤╫╘╣1⁄2╓┌║┼í╛┬φ┐╦╩2╛▌═°í┐_2009.docx]

现在，我代表市人民政府，向大会报告政府工作，请予审议，并请市政协委员和列席会议的同志提出意见。

　　一、2008年工作回顾

　　2008 年是极不平凡、极不寻常的一年。一年来，我们遭受了低温雨雪冰冻灾害的侵袭，感受了汶川大地震带来的民族苦难，经历了北京奥运会成功举办和“神七”飞天成功的喜悦，遭遇了百年不遇的全球金融危机。全市人民在省委、省政府和市委的正确领导下，在与灾害和困难的斗争中，同舟共济，奋勇拼搏，赢得了我市经济社会发展的新成就。

在过去的一年里，我市综合实力再上台阶。生产总值达到 628.3 亿元，增长 13.5% （按可比价）；财政总收入达到 68 亿元，增长 22.8% ，跃入全省第三位，其中一般预算收入 36.7 亿元，增长 14.3 ％。经济质量得到提升。财政总收入占生产总值的比重达到 10.8 ％，同比增加 0.32 个百分点；税收占财政总收入的比重达到 88.3 ％，同比增加 3.9 个百分点；三次产业结构调整为 19.1 ： 47.4 ： 33.5 ，二产同比增加 1.6 个百分点。“三驾马车”协调发展。预计完成全社会固定资产投资 515.4 亿元，增长 25.4% ，其中城镇以上固定资产投资 420.2 亿元，增长 34 ％；外贸出口总额 4.8 亿美元，增长 70 ％；社会消费品零售总额 231.5 亿元，增长 23.9 ％。民生事业成效显著。去年年初确定的就业和再就业、社会保障、社会救助、医疗保障、济困助学、群众住房、扶贫开发、改善群众生产生活条件等八个方面 46 件民生实事全部落实，促进了社会的和谐稳定。基础设施不断加强。完成了一批工业、农业、交通、公用设施等重点基础项目建设，尤其是抓住中央“扩内需、保增长”的机遇，争取到一批基础设施建设项目，为上饶今后的持续发展和跨越发展奠定了基础。

　　一年来，我们着力抓了八个方面的工作：

　　（一）着力巩固农业基础地位

　　综合生产能力稳步提高。一产完成增加值 120 亿元，同比增长 5.6 ％。粮食总产达到 57.3 亿斤，同比增长 5.3 ％，连续五年创历史新高；油料、经济作物、畜（禽）牧业、水产业等产量全面增长，农业产出效应进一步提升。

现代农业建设成效明显。成功申报省级农业标准化示范区 18 个，无公害农产品、绿色食品、有机食品开发连续 4 年居全省第一。新增省级以上龙头企业 34 家、国家级龙头企业 1 家；新增销售收入过亿元企业 6 家，新增农民专业合作社 287 家。

　　新农村试点建设扎实推进。投入新农村建设资金 8 亿元，全面完成 1330 个新农村试点村建设任务，改路 1300 多公里、改水 6.9 万户、改厕近 7 万户。农村生产生活条件进一步改善。

　　（二）着力加快工业化进程

　　园区主平台作用凸显。全市工业园区实际开发面积位居全省第一，投产工业企业 828 户；预计实现主营业务收入 630.7 亿元，同比增长 38.6 ％；实现税金 27.4 亿元，同比增长 42.8 ％；安置就业 13.3 万人。园区对财政的贡献率达到 32.3 ％。工业集约化经营成效显现。

　　主导产业地位提升。初步形成了以有色金属、光伏、机电光学和新型建材为主导产业的发展格局。四大主导产业规模以上企业实现主营业务收入 469.8 亿元，占全市规模以上工业的 77.1 ％。其中有色金属产业实现主营业务收入 232.2 亿元，占全部规模以上工业主营业务收入的 38.1 ％。产业结构调整步伐进一步加快。

重点企业支撑有力。全市规模以上工业企业达到 644 户，实现主营业务收入 609.1 亿元，同比增长 37.3 ％。其中，主营业务收入过 5 亿元企业 20 户、过 10 亿元企业 5 户。传统产业升级改造推进快、效果好。

贡献份额持续加大。全市完成规模以上工业增加值 179 亿元，同比增长 26.6 ％；实现利税总额 33.2 亿元，同比增长 21.4% ；来自工业的税收占财政总收入的比重达到 50 ％，较上年提高 2 个百分点。为促进经济增长、吸纳就业发挥了重要作用。

（三）着力提高第三产业比重

商贸物流业稳步发展。全市商贸物流业增加值达到 96.7 亿元。成交额过亿元的市场达到 31 家。 12 个县（市、区）全部成为国家商务部“万村千乡”市场工程试点县，共建设、改造标准化农家店 710 个。江南商贸城、鄱阳湖国际水产城、赣东北农副产品物流中心等重点商贸物流项目进展顺利。

旅游业加快发展。三清山成为世界自然遗产地，婺源跻身首批“中国旅游强县”，婺源灵岩洞景区被评为国家 4A 级景区，申报鄱阳湖国家湿地公园获得批准。全年接待国内游客突破 1000 万人次，旅游综合收入达到 60.8 亿元，同比增长 20.4 ％；入境旅游 6.2 万人次，同比增长 43.2 ％，创汇 1972 万美元，同比增长 62.3 ％。旅游总收入占全市生产总值的比重达到 9.7 ％，成为市域经济重要支柱产业。

金融业健康发展。去年年末，全市金融机构人民币各项存款余额 610.8 亿元，较年初增加 95.1 亿元，增长 18.4 ％；人民币各项贷款余额为 365.2 亿元，较年初增加 48.6 亿元，增长 15.4% 。

保险、邮电通讯、交通运输、烟草、石油、房地产等产业平稳发展。

（四）着力推进城镇化建设

规划工作不断加强。完成了《上饶市城市总体规划》、 省级风景名胜区总体规划、乡镇总体规划、行政村建设规划， 以及市中心城区的 一系列专项 规划的编制和修编工作； 横峰、 鄱阳、铅山、弋阳等县的城市总体规划修编工作 基本完成。 加强规划执法和规划审批后的管理，维护了规划的严肃性、权威性。

建设力度不断加大。城镇化率提高 2 个百分点，达到 33.5% 。全市投入城市建设资金 65 亿元，同比增长 16.1 ％。市中心城区开工建设城建重点项目 22 个，总投资达到 32.6 亿元。其中，行政中心大楼、服务中心、会议中心、市民公园、广平街、锦绣路等工程已建成投入使用，叶挺大道、信江大桥完成改造，高速公路东、西出口，三清山东大道、凤凰西大道建成通车，双塔公园二期、紫阳公园、旭日公园如期完工，一批里弄小巷得到改造。县城建设投入加大，一批重点工程相继开工建设。全市城区新建改造绿地面积 391.9万平方米，中心城区绿地率达到36.3 ％，绿化覆盖率达到 41.3 ％。上饶展示出靓丽的城市形象，成为赣浙闽皖四省交界区域内最适宜居住和创业的城市之一。

城镇管理不断规范。中心城区深入开展了以整治“十乱”为重点的城市环境综合治理工作，市容市貌得到改观。村镇人居环境加快改善，累计完成投入 43.9 亿元，同比增长 10 ％。全市建有全国重点镇 9 个、省重点镇 27 个、全省后备示范镇 1 个、国家历史文化名镇名村 2 个，省级历史文化名镇名村 14 个。

（五）着力增强发展的内在活力

各项改革继续深化。市县两级机关事业单位资产管理体制改革取得良好成效；医药商业企业改制工作顺利推进，朝阳磷矿政策性破产和招商工作迈出实质性步伐，省属江西有色金属冶炼加工总厂和弋阳江冶有色金属加工厂划转地方管理顺利交接。林业产权制度主体改革和国有农场税费改革取得阶段性成果，林业产权制度配套改革稳步推进。

开放型经济势头良好。外贸进出口总额达到 5.6 亿美元，增长 84.6 ％。实际利用外资 3 亿美元，增长 15 ％；现汇 1.5 亿美元，现汇率达到 50 ％；实际引进省外 5000 万元以上工业项目资金 105 亿元，增长 24.7 ％。南昌海关上饶办事处开关运行；上饶“无水港”建设进展顺利。对外合作领域进一步拓展。

非公有制经济健康发展。 全 市新增个体户 1.6 万户，新增私营企业 1800 多户，从业人员 55 万人，注册资本达到 252 亿元；非公有制经济增加值占生产总值的比重达到 59.6% ，较上年提高了 3.3 个百分点。全市经济结构正在形成多元发展、多极增长的新格局。

（六）着力提升综合承载能力

交通基础设施进一步改善。全市完成交通基本建设投资 11.7 亿元。上德战备公路建成通车，峰福铁路电气化改造开工建设。农村公路完成路面硬化 2552 公里 ，完成渡改桥项目 6 个，新增通水泥（油）路行政村 647 个，行政村通水泥（油）路率达到 77.2 ％，通班车率达到 91 ％。

农田水利基础设施进一步夯实。累计开工二期病险水库除险加固 761 座、完工 727 座。开工建设列入国家专项规划的病险水库除险加固工程 69 座。伦潭水利枢纽工程开工建设。滨湖地区排涝站更新改造工程进展顺利，鄱阳沿河圩和余干枫富联圩除险加固工程建设扎实推进。灌区和水利血防项目将于今年汛前全部完工。

能源和信息化建设进一步加强。改造和新建 11 万伏以上变电站 9 座，其中，信州 50 万伏变电站和园区 11 万伏变电站建成投入营运；铅山高店水库建成发电；灾后受损电网恢复重建工作成效明显。信息化建设不断加快，政务、企业、农村和公共领域的信息化建设顺利推进， 99 ％以上的行政村通上宽带，互联网用户接近 20 万户；所有自然村通上电话，固定电话总数突破 100 万户； 30 户以上的自然村基本实现移动信号“全覆盖”，移动用户总数达到 160 万户。

（七）着力推进社会建设

群众生活继续改善。市区居民人均可支配收入达到 12676 元，农民人均纯收入达到 4353 元，分别增长 11.8% 和 11.6% ；年末城乡居民储蓄存款余额 439 亿元，同比增长 18.6% 。部分群众就业难、读书难、看病难、住房难等问题得到有效缓解。

社会事业协调发展。荣膺全国未成年人思想道德建设先进城市，高考获得 2000 年以来最好成绩，义务教育均衡发展迈出大步，高等教育质量进一步提高，职业教育发展加快，民办教育不断规范。群众性文化活动丰富多彩；第三次全国文物普查进展顺利，非物质文化遗产保护进一步加强；文化市场管理进一步规范，影视剧创作实现“零”突破。公共卫生服务体系进一步完善，手足口病得到较好防治，问题奶粉患儿得到积极救治，爱国卫生运动深入开展。群众体育活动取得良好成效。低生育水平继续保持稳定，出生人口性别比综合治理取得成效，计划生育奖励、扶助优惠政策惠及面不断扩大，新农村、新家庭生育文明创建逐步推进，流动人口计划生育区域合作创新发展，人口出生率为 13.7 ‰，自然增长率控制在 8 ‰以内。慈善和红十字事业稳步推进，募集雪灾救灾款物 200 多万元，为汶川地震灾区募集款物 6300 多万元。

社会环境安全稳定。深入开展“平安上饶”创建活动，不断加强社会治安综合治理，有效形成了社会治安打防控一体化长效机制，社会治安明显好转。高度重视人民群众来信来访，健全和完善处理信访突出问题和群体性事件联席会议制度，深入开展县委书记、县长大接访活动，及时处理了一批复杂、疑难信访案件，妥善化解了一批影响社会稳定的群体事件，确保了北京奥运会期间全市社会政治稳定。全面加强安全生产监督管理，实现事故总起数和死亡人数“两位数下降”，安全生产形势总体平稳。着力打击非法传销活动。不断强化市场监管，全市没有发生恶性食品安全事故，玉山县荣获“国家级食品安全示范县”称号。不断健全和完善应急预案体系、管理体系和管理机制，继续加强国防教育和民兵预备役建设，在人民解放军和武警官兵的大力支持下，全市人民众志成城，取得了抗击低温雨雪冰冻灾害的全面胜利，防灾减灾体系进一步健全，应急管理能力不断提高。继续加强市长热线工作， 市长热线办连续四年被共青团中央评为“全国青年文明号”。

与此同时， 新闻出版、地方志、档案、民族、宗教、保密、外事侨务、涉台事务工作得到新加强，气象服务、防震减灾、“双拥”工作、人民防空等工作取得新进步，社会福利、妇女儿童、老龄、残疾人等事业实现新发展。

环境保护和治理力度加大。围绕鄱阳湖生态经济区建设，深入开展了水污染治理、保护“母亲河”、绿色生态建设、生态园区建设、生态城乡建设、农村清洁能源、血防综合治理、节能减排、城乡垃圾集中处理、污染物零排放等“十大行动”。围绕推进节能减排工作，淘汰关停了一批工艺落后、污染严重、无法治理的企业，否决了 39 个不符合国家产业政策或存在污染隐患的项目。不断加大监管力度，开展了第一次全国污染源普查和市重点污染源、饮用水源保护、节能减排等专项检查；在全省率先建立市级环境监控中心，对重点污染源实施在线监控。围绕环境保护和治理，全面开工建设 10 个县（市）的城市生活污水处理厂，其中万年污水处理厂在全省率先建成投入营运；市区生活垃圾无害化处理场一期工程建成投入营运；大力开展水土保持和河道采砂执法整治，完成水土流失综合治理 38 万亩；进一步强化功能区水质监测和城市饮用水水源地保护，饮水安全得到保障，地表水达标水质断面为 93.8 ％。全面启动造林绿化“一大四小”工程，造林 64.8 万亩；大力推进森林公园建设，全市建有 8 个国家级森林公园、 7 个省级森林公园。市城区环境空气质量优良率达到 100 ％。

（八）着力加强政府自身建设

自觉接受人大及其常委会的法律监督、工作监督和政协的民主监督，广泛听取各民主党派、工商联、无党派人士以及各人民团体的意见和建议。认真办理人大代表建议和政协提案，具体承办建议和提案 272 件，办复率 100 ％，满意率 95.1 ％。深入开展民主评议政风活动，积极推进阳光政务，政府部门的作风进一步改善，行政执法行为进一步规范，办事效率进一步提高。加强信用体系建设，发展环境进一步改善，荣获“ 2008 浙商（省外）最佳投资城市成功奖”、“中国粤商最佳投资城市”称号。廉政建设力度不断加大，行政监察和经济责任审计进一步加强；行政复议工作水平不断提高，普法宣传教育深入开展，法治环境建设得到进一步加强。

各位代表，过去的一年，面对复杂多变的发展环境，我们在省委、省政府和市委的正确领导下，超前谋划，主动应对，抢占先机，全市经济社会继续保持了良好的发展势头。成绩确实来之不易。这是努力实践科学发展观的结果，是各县（市、区）、各部门密切配合、同心协力的结果，是全市广大干部群众弘扬方志敏精神，团结一心、共同奋斗的结果。在此，我代表市人民政府向所有参与、关心、支持上饶建设和发展的同志们、朋友们表示崇高的敬意和衷心的感谢！

在总结成绩的同时，我们也清醒地认识到，上饶仍然处于“打基础、建平台、增后劲”的发展阶段，发展不足、发展不快、发展不优仍然是我市经济社会发展的主要矛盾；总量偏小、人均偏低仍然是我市的基本现状；工业化、城镇化相对滞后仍然是制约发展的主要瓶颈；“三农”问题仍然是我市经济社会发展中不容忽视的根本性问题；保护生态、促进可持续发展仍然是我们责无旁贷的重要任务；发展民生事业、促进社会和谐仍然任重道远；提高驾驭经济工作的能力、破解难题的能力、为人民服务的能力、管理社会的能力仍然需要我们付出更艰苦的努力。特别是受金融危机的冲击，经济社会发展的不稳定、不确定因素增多，保持经济社会平稳快速发展的压力加大。对此，我们将高度重视，认真研究，努力加以解决和提高。同时，也诚恳地接受各位代表和社会各界的监督与批评，进一步做好政府的各项工作。

二、2009年工作思路

2009 年，将是新世纪以来经济发展面临困难最大、挑战最严峻的一年，也是蕴含重大机遇的一年。我们坚信：挑战蕴含机遇，困难激发潜能，压力催生动力。中央和我省相继出台一系列保增长、扩内需、调结构的政策措施，为促进产业转型升级、完善基础设施、实施民生工程创造了重要条件。只要我们把思想和行动统一到市委二届五次、六次全会的精神上来，坚定信心，冷静思考，积极应对，扎实工作，善于从变化的形势中捕捉发展机遇，在逆境中发现和培育有利因素，坚持用发展的办法解决前进中的困难和问题，就一定能够战胜各种挑战，保持经济平稳快速发展，开拓新的更加广阔的发展空间。

按照市委二届五次、六次全会精神，今年政府工作的指导思想是：坚持以科学发展观为指导，深入贯彻落实党的十七大、十七届三中全会、中央经济工作会议和胡锦涛总书记在江西视察时的重要讲话精神，深入贯彻落实省委十二届八次全会和市委二届五次、六次全会精神，坚定不移地落实“保增长，扩内需，调结构”的宏观政策，坚持“掉头向东、通江达海”的开放战略，坚定“建设四省交界区域中心城市”的目标，紧紧围绕打造“一个枢纽”、建设“四个基地”的发展格局，围绕保增长、保民生、保稳定，全力实施“六大”举措，变压力为动力，化危机为生机，变经济波动期为发展机遇期，努力保持经济社会持续平稳快速发展，各项经济指标和各项工作都要瞄准全省第一方阵，争先进位。力争在 2009 年，提前一年完成“十一五”规划主要经济指标；到 2010 年，撤地设市十周年之际，财政收入超过 100 亿元；到 2011 年，本届政府任期届满时，生产总值超过 1000 亿元。

按照上述要求，今年全市经济社会发展主要预期目标是：生产总值达到 725 亿元，增长 13 ％；财政总收入 80 亿元，增长 17.6 ％；固定资产投资增长 30 ％；社会消费品零售总额增长 20% ；城镇居民人均可支配收入增长 10 ％，农民人均纯收入增长 8 ％；居民消费价格总水平涨幅控制在 4 ％左右；人口自然增长率控制在 8 ‰以内；城镇登记失业率控制在 4.5 ％以内；城镇化率提高 2 个百分点；每万元生产总值能耗、电耗、化学需氧量、二氧化硫排放量严格控制在省政府下达的指标之内。

为实现上述目标，我们要重点抓好以下八个方面的工作：

（一）大力实施“双千”工程，全力以赴保增长

保增长是今年的首要任务。围绕保增长的目标，从今年起，在全市深入开展“双千工程”（实施一千个以上重点项目、落实千亿元以上投资）。其中，今年预期安排新建、续建、建成各类项目 755 个，年内计划完成投资 538.2 亿元。

一是围绕强化产业支撑推进一批项目建设。 预期安排建设 197 个项目，本年度计划完成投资 180.4 亿元。在有色金属产业上，重点抓好上饶华晟年产 4 万吨铜、 5000 吨钴，和丰铜业年产 12 万吨再生阳极铜、 10 万吨电解铜，金德铅业年产 20 万吨铅、20万吨锌， 弋阳富达铜材年产 1400 万套水龙头及水暖配件等项目。在光伏产业上，重点抓好江西晶科能源年产 300 兆瓦太阳能电池、江西加华年产 200 兆瓦多晶硅、拓普公司年产 1000 兆瓦薄膜太阳能电池、江西光旭能源科技年产 250 吨单晶硅等项目。在机电光学产业上，重点抓好婺源洲际科技新型电子信息材料、江西奥得电子年产 3600 万只节能灯、江西索普信年产 1 亿只电子节能灯，江西钰成光学年产 6 亿片光学镜片、 5000 万只镜头等项目。在新型建材产业上，重点抓好弋阳海螺年产 160 万吨水泥、 177 万吨熟料，江西大自然人造板等项目。

二是围绕强化交通能源和通讯保障能力推进一批项目建设。 预期安排建设 65 个项目，本年度计划完成投资 95.7 亿元。在交通基础设施上，继续抓好峰福铁路电气化改造，力争开工建设京福高速铁路、杭南长铁路客运专线、九景衢铁路，推进皖赣铁路改造；争取上半年开工建设上武高速公路，下半年开工建设昌德高速公路，加快推进上饶三清山机场和上德高速公路前期工作；抓紧做好鄱阳港务区、信江、饶河航道整治等项目的前期工作，扎实推进 320 国道升级改造工程，全面完成年度农村公路和渡改桥建设任务。在能源建设上，力争开工建设黄金埠电厂二期工程，抓紧做好上饶赣东火电厂前期工作，争取尽快启动核电站选址工作。继续抓好城市配网和中低压电网改造，扎实推进农电“三新”发展战略。在信息化建设上，大力推进第三代移动通信技术 TD 网络建设，逐步推广互联网无线化， 继续推进国家农村信息化综合服务试点工作， 进一步提升社会信息化水平。

三是围绕强化农田水利基础设施推进一批项目建设。 预期安排建设 124 个项目，本年度计划完成投资 51.7 亿元。加快推进伦潭水利枢纽工程建设；新开工 78 座列入国家专项规划的病险水库除险加固工程；抓紧实施鄱湖灌区、七一灌区和饶丰灌区改造项目，实施余干新开河排涝总站更新改造工程；继续推进在建的鄱阳沿河圩、余干枫富联圩工程建设，实施广丰河北堤、余干大溪圩、鄱阳中洲圩工程建设，争取开工建设上饶市城市防洪三江导托渠工程；争取启动鄱阳湖二期治理工程第六个单项。

上述重大项目是上饶扩内需的抓手、保增长的关键。这些项目的建设将极大地改变上饶的发展格局，为上饶未来的发展开辟广阔的前景！

（二）切实转变发展方式，加快新型工业化进程

新型工业化是上饶快速发展的必由之路。坚定不移地实施主攻工业战略，不断加大工业投入，今年力争全部工业主营业务收入达到 1000 亿元，其中，规模以上工业实现主营业务收入达到 720 亿元。

强化园区支撑。坚持“工业向园区集中，项目向园区集聚，政策向园区倾斜”，继续完善工业园区基础设施和功能配套，进一步提升园区的综合承载能力。上饶经济开发区、广丰工业园要在全省争先进位，横峰工业园进入全省一类工业园区行列，其他县市的工业园区争取晋升为全省二类以上工业园区。大力发展生态工业，努力把工业园区建成县域经济带动区、体制和科技创新示范区、城市发展新区。确保全市工业园区主营业务收入达到 900 亿元。

壮大主导产业。积极围绕有色金属、光伏、机电光学、新型建材四大主导产业，进一步优化要素组合和资源配置，促进产业结构优化升级，构建具有上饶特色的产业集群。力争以华丰铜业、和丰铜业、金德铅业为龙头的有色金属产业，以江西晶科能源、赛德能源为龙头的光伏产业，以凤凰光学、一舟电子为龙头的机电光学产业和以弋阳海螺水泥为龙头的新型建材产业的主营业务收入达到 600 亿元，占规模以上工业主营业务收入的 80 ％以上。

扶优重点企业。进一步增强服务意识，创新服务方法，强化服务措施，最大限度地缩短企业从签约到落地建设的周期。大力推进 150 项 5000 万元以上招商引资工业项目建设，争取年内完成投资 110 亿元以上。积极帮助企业提高劳动生产率、人均创利率、资金利润率和资源能源利用率，开拓国内外市场，努力打造具有市场竞争力的大企业、大集团。力争新增规模以上企业 60 户、年销售收入过亿元企业 30 户。

加大政策支持。进一步加强农村富余劳动力职业技能培训，降低工业园区企业职工参加社会保险缴费基数，降低企业用工成本。加快口岸物流体系建设，大力支持拓展铁海联运快速物流通道；进一步清理整顿公路和城市道路收费站，有效降低企业物流成本。进一步强化要素保障，以市级融资担保机构为平台，加快县（市、区）融资担保机构组建步伐，加强产业转移企业的信用对接，大力推进银企合作，支持企业多渠道融资；推行峰谷电价政策，保障企业电力供应。继续加大财税支持力度，认真落实有关税费减免政策，依法依规兑现承诺，切实维护投资者的合法权益。

（三）积极发展现代农业，千方百计促进农民增收

农业始终是上饶发展的基础和优势。围绕增加粮食总产，发展优质农产品，提高农民收入，推进新农村建设，不断加快我市现代农业的发展。

提高农业综合生产能力。 围绕 实施“新增百亿斤优质商品粮工程”，重点推进标准粮田建设工程、高产创建示范工程、良种推广工程、造地增粮富民工程、水源和灌溉工程、农业机械化工程、农业保障服务工程、沃土工程、防灾减灾工程等方面的项目建设，改造中低产田 11 万亩。不断健全农技推广、重大动物疫病防控和农产品质量安全监管体系，确保粮食种植面积达到 810 万亩，力争粮食总产达到 58 亿斤 。

提高农业产业化经营水平。充分发挥农产品资源优势，围绕打造国家粮仓和优质农产品供应基地，加快发展现代农业。力争建设标准化优质农产品生产基地 10 个；规模以上农业企业发展到 430 家；出口创汇企业发展到 45 家，出口创汇 2.7 亿美元；粮食、畜牧产业产值突破 50 亿元。

提高新农村村点建设质量。 按照景区、湖区、郊区新农村建设的不同特点，采取分类推进的办法，抓好今年 1300 个以上新农村村点建设。不断加强村镇规划，加快农村基础设施建设步伐，切实改善农村人居环境。

（四）打造旅游商贸物流基地，提升现代服务业发展水平

旅游商贸物流为“保增长”提供了巨大潜力。充分发挥我市地理位置、综合交通和旅游资源三大优势，把现代服务业放在优先发展的位置，着力提高现代服务业对经济增长的贡献率。

大力推进旅游休闲基地建设。 继续加强旅游品牌创建，抓紧做好龟峰捆绑申报世界自然遗产、三清山申报世界地质公园、婺源古村落申报世界文化遗产拓展项目；扎实做好三清山、龟峰和婺源江湾争创国家 5A 级景区，上饶集中营、铜钹山、灵山、神农源创国家 4A 级景区，万年仙人洞和吊桶环遗址申报国家级风景名胜区的相关工作。抓紧抓好现有景区整合提升，充分发挥三清山、龟峰两大世界级品牌的龙头带动作用，加快旅游配套功能建设，力争三清山旅游集散中心和世界遗产博览中心早日开工建设。抓紧建设上饶市区一级旅游服务区和三清山、玉山、婺源等直接为景区服务的二级旅游服务区。抓好旅游商品开发、星级宾馆建设及交通等配套设施建设。继续加大市场拓展力度，稳步推进旅游管理体制改革，不断加强旅游从业队伍建设。力争实现旅游总收入 80 亿元，旅游总收入占生产总值比重达到 10 ％以上。

大力发展商贸物流业。抓紧做好城区商业发展和县级商业网点的规划编制工作。加快对江南商贸城、佳利商贸城、鄱阳湖国际水产城、赣东北粮食物流园、江南米业市场、亿升广场、润丰城市广场等市场的培育和提升。积极开展国家商务部“中华老字号”争创工作。深入推进“万村千乡市场工程”，新建农家店 700 个，力争配送率达到 40 ％以上。支持连锁超市向乡村扩张，积极构建城乡一体化的现代商贸流通网络。继续强化城乡市场监管，营造和谐安全的消费环境。力争新增过亿元市场 11 个，总数达到 42 个，实现商贸物流业增加值 120 亿元。

大力发展新兴服务业。重视培育评估、资本、产权、技术、咨询、策划等现代商务组织，有序发展现代商务业。支持发展以网络传输服务、计算机与软件服务以及数字内容服务为代表的信息服务业。不断发展科研开发、产品设计等生产性服务业。支持各类金融机构拓展业务、创新服务、加快发展；不断壮大和发展商业银行，积极引进外地金融机构，加快建设现代金融体系。稳步推进房地产业，坚决贯彻执行好促进住房消费的各项政策，合理引导房价，稳定市场预期，增强市场信心，促进房地产业规范有序健康发展。

（五）着力实施“双轮驱动”，统筹城乡协调发展

统筹城乡协调发展是科学发展的基本要求。围绕中心城市大建设、县域经济大发展，进一步加大 统筹城乡发展力度。预期 安排城市建设项目 216 个，年内计划投资 143.9 亿元。

加快提升城市综合服务能力。按照“提升老城区，做旺中心区，做热开发区，拓展城东区，做靓三江口”的发展思路，在提升老城区上，坚持多做“减法”，少做“加法”，重点围绕控制疏解，加强老城区停车场、地下管网、绿化、环卫等设施配套，重点改造主要节点的景观，切实改善老城区的人居环境。在做旺中心区上，围绕聚集人气，加快中心区的基础设施建设，提升中心区的公共服务功能，加强中心区示范性幼儿园、优质小学、中学，以及医院、商业、宾馆、酒店等配套功能，使中心区逐步成为上饶市的行政中心、商务中心、生态中心和文化中心。在做热开发区上，按照把开发区建成城市新区的理念，加快道路管网等基础设施建设，注重各种配套功能完善，把开发区做成一、二、三产协调发展的城市新区。在拓展城东区上，加强杭南长铁路客运专线和京福铁路枢纽与城市规划的对接。加快城东区与中心城区道路、管网的互联互通，重点建设综合性现代交通枢纽和发展商贸物流产业，迅速提升上饶对外开放的形象和水平。在做靓三江口上，围绕打造“三江六岸”核心城市景观，实施三江南岸景观改造、三江口建筑物天际线改造和亮化改造，实施滨江路景观综合改造，加快推进三江口关键节点、核心部位的景观建设，逐步构建以地标性建筑为代表的现代中心城市形象。通过 2-3 年的努力，切实增强中心城区的承载能力、集聚能力、带动能力和辐射能力。

坚持建管并重，巩固“五城同创”成果，强化综合执法，进一步加大“十项整治”力度，使市容市貌有明显变化。突出抓好城市交通整治，优先发展公共交通，着力形成有序、通达、顺畅的交通秩序。突出抓好城市违章建筑的整治，维护规划的权威性和严肃性。围绕建立长效管理机制，强化条块结合、密切协同作战，加快建立市、区、街道、居委会四级管理构架。进一步创新管理机制，落实管理责任，推行数字化管理，规范城市管理标准，全面提升城市管理水平。

加快推进县域经济发展。 以县域优势资源为依托，积极引导县域经济 向特色型转变 ，加快资源优势向经济优势转化，不断做 大县域经济总量。 进一步下放审批权限和简化审批手续，积极推行“一站式审批”、“并联审批”、“联合审批”和“网上审批”，规范项目管理，提高工作效率，支持县域经济加快发展。不断 加大县城和重点镇建设力度，加快垃圾和污水处理、绿化等基础设施建设，同步实施“创五城”战略，以县城和重点镇的建设带动县域经济加快发展。

全力做大市本级经济。 重点抓好上饶经济开发区扩园升级和申报国家级开发区工作，使上饶经济开发区成为全市工业园区的领头羊、全省工业园区的排头兵。力争今年市本级财政收入过 10 亿元；上饶经济开发区工业主营业务收入达到 220 亿元，税收突破 9 亿元。

（六）继续深化改革扩大开放，切实增强发展活力

改革开放是战胜困难、加快发展的重要法宝。围绕增强发展活力，全面推进各项改革，努力提高开放水平，建设良好的创业环境和投资环境。

进一步推进各项改革。继续加强机关事业单位国有资产监管，充分发挥国有资产对市重大建设项目的融资作用。大力发展资本市场，继续推进上饶汽运、江西远泉、三清旅游、万年贡米等企业的上市申报工作。认真实施增值税转型改革，落实好燃油税费改革的各项政策，继续推进国有农场税费改革及综合配套改革，不断深化医药卫生体制改革，探索公立医院运行机制改革。稳定和完善农村基本经营制度，认真落实稳定土地承包关系的各项措施，加快建立土地承包经营权流转市场服务体系，促进农村土地经营集约化、规模化；稳妥推进农村综合改革，加快发展农民专业合作社。继续实施好公务员法的各项配套法规，不断深化事业单位人事制度改革。

进一步强化产业招商。突出我市产业特色和比较优势，创新招商方式，明确招商重点，围绕光伏、有色金属、机电光学、新型建材、旅游、农业等重点产业，大力开展产业招商。注重引进与主导产业关联度高、带动性强、发展前景好的重大项目，积极引导外资投向现代服务业、基础设施、社会事业等领域。千方百计在引进中国 500 强、世界 500 强企业来饶投资方面实现突破。力争引进省外单项投资 5000 万元以上工业项目实际进资突破 110 亿元，其中来自重点产业的资金突破 100 亿元；实际利用外资突破 3.3 亿美元。

进一步壮大外经外贸。积极培育外贸生产型出口骨干企业，狠抓出口品牌建设，努力转变外贸增长方式；认真落实相关优惠政策，增强出口后劲。充分发挥商检和海关服务开放型经济的作用，加快建设“无水港”，确保 3 月底建成使用。抓紧做好上饶出口加工区申报工作。加强对外劳务合作，推动劳务经济健康、有序发展。实现外贸进出口突破 6 亿美元。

进一步发展非公有制经济。坚持外商内商一样重视，外资内资一视同仁，全民创业和招商引资一同推进，毫不动摇地鼓励、支持和引导非公有制经济大发展、快发展。重点支持一批有竞争力的非公有制企业做大做强，大力支持我市民营企业与中央省属国有大型企业联姻，推动非公有制经济总量上规模、结构上层次、质量上水平、管理上台阶。鼓励外出务工经商人员返乡创业，培育一批新的经济增长点。协调解决非公有制经济发展中出现的各种问题，依法保护非公有制经济的合法权益。

（七）大力推进民生事业，加快构建和谐上饶

搞好民生建设既是扩大内需也是建设和谐社会的需要。必须在加强社会保障，改善群众生产生活条件，繁荣各项社会事业上迈出新步子，努力促进人的全面发展和社会全面进步。

一是不断完善社会保障体系。 坚持就业优先， 重点办好进一步扩大就业、加快农村富余劳动力转移、加强就业培训、创业指导、提供小额贷款、帮助残疾人就业等实事，全力以赴抓好城市新增劳动力、返乡农民工和大学毕业生的就业工作。扩大保险覆盖面，重点办好提高企业离退休人员养老金水平、扩大养老保险覆盖范围、推进被征地农民养老保险试点、提高失业保险统筹水平、扩大城镇职工工伤保险覆盖范围等实事。强化社会救助，重点办好提高城乡低保对象补助标准，提高农村五保户供养标准，完善国有企业“老工伤”人员伤残津贴制度和提高上世纪 60 年代精简下放老职工救济金水平，提高部分困难企事业单位退休职工生活补助标准、妥善安置破产改制企业职工，提高城镇“三无”特困群众供养标准，建立农村困难群众住房救助制度，完善城乡临时救助制度和改善孤残儿童、流浪未成年人的生活条件，继续实行农村计划生育奖励制度，建立因自然灾害死亡人员抚慰金制度，规范提高义务兵优待金标准和退役士兵自谋职业补助水平，加强对困难群众的法律援助等实事。

二是加强卫生医疗体系建设。 抓好市精神卫生中心、农村卫生院等重点卫生项目的建设工作，不断完善重大传染病预防控制体系，进一步加强各类突发公共卫生事件应急能力。重点办好扩大城镇职工基本医疗保险覆盖范围，推进城镇居民基本医疗保险制度，健全新型农村合作医疗制度，完善城乡困难群众大病医疗救助制度，扩大全市儿童一类疫苗免费接种范围，发放公共卫生服务卡，积极帮助残疾人康复，完善优抚对象看病就医制度，完善乡镇卫生院人员经费保障机制，对村级医生承担公共卫生服务给予津贴补助，加强食品药品安全检测，强化农村三级卫生服务网络和城市社区卫生服务体系建设等实事。

三是积极发展科技、教育、文化、广电、体育事业。 在科技方面， 重点抓好市科技公共服务平台建设，筹划组建一批市级重点实验室或研究中心，继续抓好省级农业科技园建设。在教育方面，增加城乡义务教育发展经费，完善义务教育保障机制，继续实施好农村义务教育均衡发展工程、农村留守儿童关爱工程，扎实做好农民工子女的就读工作，切实规范民办教育，进一步巩固义务教育发展成果；抓好城市中小学规划建设，筹建上饶中学和上饶特殊教育学校，推进农村中小学校舍建设工程；继续支持上饶师范学院建设，大力发展职业教育，加快上饶卫校新校区建设，积极筹建医学高等专科学校；扩大家庭经济困难学生资助范围，资助城乡困难家庭子女免费入读技工院校，专项补助财政困难县（市、区）义务教育绩效工资和边远地区教师发放特殊津贴，进一步满足高校经济困难学生助学贷款需求。在文化方面，抓紧筹办第六届赣浙皖闽四省四市民间艺术节，积极参加第四届全省艺术节，切实加强非物质文化遗产项目及传承人的申报和保护工作，尽快完成上饶市图书馆建设，加快建设信江书院环境景观工程，进一步加强文化市场监管，免费向公众开放博物馆和纪念馆；加强社区文化广场、乡镇综合文化站、基层文化阵地和“农家书屋”建设，深入开展农村文化“三项活动”。在广播电视事业方面，加快建设上饶艺术中心，积极推进 821 台整体搬迁，不断提高行政村宽带网络覆盖率，大力推进村村通广播电视工程，帮助 20 户以上已通电自然村全部通广播电视。在体育事业方面，举办第二届市运会，备战好省运会，对基层群众体育健身场所建设予以补助，扎实开展全民健身活动。

四是切实改善人民群众生产生活条件。 加大保障性住房建设力度。 重点办好廉租住房、经济适用住房建设，推进棚户区（城市危旧住宅区）改造等实事，建设经济适用住房 3367 套，廉租住房 3106 套。其中，中心城区经济适用住房建设 1000 套，总计 7 万平方米 ；廉租住房 700 套，改造棚户区危旧房面积 30 万平方米。着力推进扶贫开发。重点办好帮助全市 610 个扶贫开发工作重点村实施村级扶贫规划，完成 6000 人移民搬迁任务，积极开展产业化扶贫等实事。改善农村生存环境。重点办好解决 19.8 万农村人口的饮水安全问题，新增农村沼气用户 2 万户，加强农村家庭生活垃圾处理，扩大农业政策性保险试点范围，提高生态公益林补偿标准，进一步保障农村居民用电，加强农村交通基础设施建设等实事。

五是高度重视人口和计划生育工作。 严格落实“一票否决”制度，全面推行出生实名登记制，加大“两非”整治力度，积极推行计划生育村民自治，不断完善利益导向机制，继续抓好新农村、新家庭生育文明创建活动，着力提高计划生育率，促进人口和计划生育事业造福人民，惠及子孙后代。

六是全力维护社会安全稳定。狠抓安全生产。进一步规范企业生产和经营秩序；全面落实安全生产“一岗双责”制；强化企业安全生产主体责任，严格执行安全生产责任追究制；进一步深化重点行业和重点领域安全生产专项整治，狠抓安全隐患的排查治理，有效遏制重特大事故发生。

加强社会治安综合治理。严厉打击各类违法犯罪活动，进一步完善群防群治网络，全面提高社会面防控能力，让群众的安全感明显提高。扎实推进普法工作。重视和加强信访工作，进一步畅通诉求渠道，积极引导群众通过合理途径理性反映和解决利益诉求；继续坚持领导接访、包案下访制度，健全矛盾纠纷排查调处工作机制；切实解决农民增收、企业改制、劳动就业、劳资纠纷、征地拆迁以及教育、医疗等群众普遍关注的热点问题，努力化解社会不稳定因素，促进社会和谐。

不断完善应急管理体系。继续完善全市应急管理组织体系建设，强化公共安全应急处理机制，健全疾病预防控制网络和食品、药品安全监管体系，发挥应急管理职能，及时稳妥地处置好各类突发公共事件，确保全市大局稳定。

加强国家安全、民兵预备役、“双拥”、国防动员和民族宗教工作，切实关心老龄、妇女、儿童和青少年事业。

（八）加强环境保护治理，建设绿色生态上饶

优美的生态环境是上饶发展的宝贵财富。从节能减排、防治污染、加快绿化等入手，切实保护生态环境，加强生态环境建设，为经济社会发展提供良好的生态环境。

以推进新技术运用为重点，突出抓好节能减排。严格实行节能减排目标责任制。将年度目标任务量化细化分解落实到各地、各重点行业和企业，责任到人，督办到位。严格控制“三高”行业的项目和投资，新上项目要严格执行环境影响评价和“三同时”制度，把好项目准入关。突出抓好重点地区、重点行业和重点企业的节能减排。抓好建筑节能，大力推广使用节能减排技术，加快电力、钢铁、水泥、化工等行业的重点企业脱硫设施改造，抓紧抓好 LED 蓝绿光芯片、 LDK 太阳能电池及组件等 25 个节能项目续建，新开工 3 个节能减排和资源综合利用项目，力争开工建设窑炉改造、余热余压利用、尾矿和废渣利用等 15 个项目，建成华丰铜业 6 万吨 IT 电路板和污泥处理等资源综合再利用项目。努力建设科学合理的资源利用体系，切实提高资源利用效率。

以水污染防治为重点，不断加强环境综合治理。依法加强水资源利用管理与水质保护，确保饮用水源安全。继续加大水土流失治理力度，确保完成水土流失综合治理 38 万亩。进一步加强河道湖泊管理力度，严格涉河建设项目审查审批。强化信江、饶河源头保护，重视抓好信江、饶河等主要干流的水质保护。构建全市环境监察、监测网络，不断加强环保能力建设。加大对重点污染企业、尤其是存在污染事故隐患企业的监管力度，防止环境污染事故发生。积极开展农村环境综合治理，推进环境优美乡镇、生态县（市）创建；不断加强城区环境综合整治，努力建设环境优美城市。

以鄱阳湖生态经济区建设为契机，继续加强生态环境建设。 深入推进“一大四小”造林绿化工程 ， 造林 75.8 万亩，完成五年规划 40 ％的造林任务；继续抓好生态公益林、退耕还林、长防林、血防林项目建设，深入实施三清山重点火险区治理项目 。加快实施我市鄱阳湖周边农村新能源工程、环湖生态林业工程等一批重大生态环境工程。加快鄱阳湖湿地公园、槠溪河 生态湿地公园建设进程，开工建设云碧峰森林公园保护防治及配套设施工程 ，力争新建 2 个省级以上森林公园和 2 个省级自然保护区。

三、进一步加强政府自身建设，倾力打造人民满意政府

应对当前的复杂形势，完成新一年的艰巨任务，要求各级政府必须进一步加强自身建设，切实转变工作作风，着力提高运行效率。

一是加强学习，增强本领。 当今时代，科技日新月异，市场瞬息万变，发展风起云涌。我们面临最大的恐慌是知识的恐慌，最大的挑战是能力的挑战。各级政府工作人员只有认认真真地学习，才能丰富我们的知识，只有持之以恒地学习，才能提高我们的能力，跟上时代的进步。要深入学习领会邓小平理论、“三个代表”重要思想的内涵和精髓，坚持以科学发展观为统领，始终坚定正确的政治方向，切实增强贯彻落实中央重大决策的自觉性；深入学习市场经济理论，深化对市场运行规律的认识，不断提高驾驭市场经济的能力；深入学习做好本职工作必需的知识，不断增加知识储备，提高自身综合素质。坚持理论联系实际，学以致用，把学习的成果转化成各级政府解决问题、破解难题、推进工作的实际能力，把学习的成果转化为推动上饶经济社会又好又快发展的现实成效。

二是依法行政，履职尽责。 各级政府要严格按照法定权限和程序行使权力、履行职责。自觉接受人大及其常委会监督，认真落实人大及其常委会的决定，及时向人大及其常委会报告工作。积极支持人民政协履行政治协商、民主监督、参政议政的职能，主动听取各民主党派、工商联、无党派人士及各人民团体的意见和建议。着力提高人大代表建议和政协提案的办理质量。进一步推进政务公开，提高决策透明度。不断健全公众参与、专家论证和政府决定相结合的行政决策机制，坚持重大问题集体决策和重大决策征求意见制度，推进决策科学化、民主化。认真做好行政复议，及时纠正执法偏差，有效化解行政争议。致力打造在党委领导下、不折不扣执行上级决策的人民政府，打造在人大依法监督、政协参政议政下的人民政府，打造聚精会神抓发展、全心全意为人民谋利益的人民政府。

三是转变职能，提高效率。 坚持“市场出效率、政府保公平”的原则，加快政府职能转变。把政府该管的事努力管好，把不该管的事全面放活，把财力和物力更多地集中到推进社会发展、解决民生问题上来，把精力和注意力更多地集中到促进社会公平正义、维护社会秩序和稳定上来。大力开展“机关效能年”活动，继续加强政风行风监督。进一步深化行政审批制度改革，大力推行首问负责制、限时办结制、超时默许制，坚决整治行政不作为、慢作为和乱作为的现象，着力提高机关办事效率。进一步优化政务环境，牢固树立“发展经济靠企业家，优化环境靠政府”的理念，努力拓展服务空间，对企业和基层要求办理的事项，可多可少的程序要少，可收可不收的费不收，着力把上饶打造成效率最高、服务最优、成本最低、环境最好的投资创业区域。进一步畅通民情通道，大力加强市长热线等政府与群众联系沟通平台的建设，自觉接受新闻舆论监督和社会监督，倾听群众诉求，切实解决人民群众反映强烈的问题。

四是改进作风，务实作为。 作风好坏，决定事业兴衰，关系民心向背。改进作风，落实是重点、责任是关键、效率是保证。各级政府都要做到，党委有决策，政府就要见行动；群众有呼声，政府就要有作为。要围绕市委的决策，全力以赴抓落实；围绕企业的要求，积极主动抓落实；围绕群众的呼声，扑下身子抓落实。进一步完善目标管理机制、任务分解机制、工作推进机制、绩效考核机制和责任追究机制，真正形成一级抓一级、层层抓落实的工作格局，合力推进各项工作。要时刻牢记，政府的权力是人民赋予的，人民是我们的衣食父母。政府的工作人员一定要摆正自己的位置，坚持“权为民所用，情为民所系，利为民所谋”。各级政府越是在困难时期，越要把群众的冷暖安危挂在心上，想群众之所想，急群众之所急，多为群众办好事、办实事，让上饶的百姓得到更多实惠。

五是廉洁从政，干净做事。 各级政府工作人员都要严格遵守廉政建设的各项规定，建立健全教育、制度、监督并重的惩治和预防腐败体系，严厉查处腐败案件，从源头上预防和遏制腐败。今年各个领域的投资力度大，建设项目多，各级政府及其部门更要依法依规办事，实施阳光操作；更要加强管理，规范资金使用，确保资金安全；更要强化审计监督，及时发现偏差，坚决予以纠正。各级政府及其部门的领导既要管好自己，又要管好分管领域和身边的人，自觉在每个环节上自律，从点滴细微处防范，明明白白决策，干干净净做事，堂堂正正做人，努力实现“经济健康发展、干部健康成长”的“双健康”目标。

各位代表，上饶的发展正处在一个关键时期，困难和挑战考验着我们，责任和使命激励着我们。让我们更加紧密地团结在以胡锦涛同志为总书记的党中央周围，在中共上饶市委的坚强领导下，大力弘扬方志敏精神，进一步坚定信心、和衷共济，在推进上饶科学发展、和谐发展、平稳快速发展的道路上阔步前进！
